# ‘How would you call this in English?’ Being reflective about translations in international, cross-cultural qualitative research

**DOI:** 10.1007/s40037-017-0329-1

**Published:** 2017-02-20

**Authors:** Esther Helmich, Sayra Cristancho, Laura Diachun, Lorelei Lingard

**Affiliations:** 1 Center for Education Development and Research in Health Professions, University of Groningen, Groningen, The Netherlands; 2 Centre for Education Research & Innovation and Department of Medicine, Schulich School of Medicine & Dentistry, Western University, London, Ontario Canada

**Keywords:** Translations, International collaboration, Qualitative research

## Abstract

**Introduction:**

Medical education researchers increasingly collaborate in international teams, collecting data in different languages and from different parts of the world, and then disseminating them in English-language journals. Although this requires an ever-present need to translate, it often occurs uncritically. With this paper we aim to enhance researchers’ awareness and reflexivity regarding translations in qualitative research.

**Methods:**

In an international study, we carried out interviews in both Dutch and English. To enable joint data analysis, we translated Dutch data into English, making choices regarding when and how to translate. In an iterative process, we contextualized our experiences, building on the social sciences and general health literature about cross-language/cross-cultural research.

**Results:**

We identified three specific translation challenges: attending to grammar or syntax differences, grappling with metaphor, and capturing semantic or sociolinguistic nuances. Literature findings informed our decisions regarding the validity of translations, translating in different stages of the research process, coding in different languages, and providing ‘ugly’ translations in published research reports.

**Discussion:**

The lessons learnt were threefold. First, most researchers, including ourselves, do not consciously attend to translations taking place in international qualitative research. Second, translation challenges arise not only from differences in language, but also from cultural or societal differences. Third, by being reflective about translations, we found meaningful differences, even between settings with many cultural and societal similarities. This conscious process of negotiating translations was enriching. We recommend researchers to be more conscious and transparent about their translation strategies, to enhance the trustworthiness and quality of their work.

## Introduction

Exploring the pressing problems of medical education often calls for research in multiple international contexts and cultures [1, 2]. As a consequence, medical education researchers collect data in different languages, but they will commonly use only one language, typically English, to collaborate and communicate in the international scientific community. Therefore, medical education research implies an ever-present need to translate, both in cross-cultural teams and in teams where research is translated from its original language for dissemination in English. However, this translation from the original language into English often occurs uncritically, providing ‘perfect’ quotes as if the participants had shared their insights in English. Such translation strategies are very rarely described in published work, which may undermine the trustworthiness and transferability of study results.

Although translation challenges tend not to be explicitly addressed within the major health professions education journals, there is a body of knowledge to build upon in the broader scientific literature, most prominently from anthropology and the interdisciplinary field of translation studies. Translation takes place when language is being converted from one (source) language into another (target) language [3]. By this process, an accurate translation should be reached, both technically and conceptually [4]. Conceptual equivalence is important since literal translations in the target language do not always express the essential meanings of the source language [5].

Translation can be needed at different stages in the research process: A) prior to data collection (e. g. developing the interview guide or questionnaire) [6]; B) at data collection (e. g. during real-time conversation with participants) [7]; C) during data preparation (e. g. translating transcripts) [8]; D) during data analysis (e. g. translating codes/themes) [9]; and E) at dissemination of findings (e. g. translating quotes) [10]. The focus in this paper will be on the different challenges researchers may face during the translation of qualitative data as a preparation for collaborative data analysis in an international team, translating in the process of analyzing qualitative data in different languages, and translating during the writing up of the final results.

We seek to spark increased interest in and enhance researchers’ reflexivity regarding translations in qualitative research. The paper has two parts. First, we share some of the specific challenges we encountered in a recent international (Netherlands-Canada) qualitative research collaboration regarding to translations. Second, we relate our experiences to what is known about translations from the literature about cross-language or cross-cultural research.

## Translations in an international cross-language/cross-cultural study

### Description of the study

In an international study of medical trainees’ experiences of complexity in clinical practice, we carried out interviews in both Canada and the Netherlands. Canadian participants were interviewed in English, and Dutch participants in Dutch. The research team consisted of medical education researchers with different professional backgrounds: two physicians working in elderly care medicine (EH, LD), a soft systems engineer (SC), and a rhetorician (LL). Two members of the team were native English speakers from Canada (LD, LL), and two researchers had a different first language, both of whom are socio-linguistically (referring to a combination of social, technical and cultural language abilities) and strategically (referring to the ability to deal with unfamiliar words or slang terms) competent in English as their second language (EH, first language Dutch; SC, first language Spanish) [11].

To allow joint data analysis, we translated parts of the Dutch interviews into English; this was done by a Dutch medical student in close collaboration with the first author (EH). Throughout the translation process, EH copied all salient parts of the transcripts into a table. By salient, we mean instances where translation could be challenging or a matter of debate, or instances where translation could be particularly relevant to the focus of our study. EH provided the original text in Dutch, and a literal and/or more conceptual translation in English, together with comments or justifications regarding the translation. One of the Canadian researchers (LD) responded to these translations on multiple occasions, suggesting other possible translations, or offering information from her Canadian background. This was followed by a team discussion [5], in which the four of us discussed the translations and the reflections from both Dutch and Canadian perspectives, seeking other possible translations, playing with words and cultural notions, until we arrived at a shared understanding of the words, the concepts, and the differences and similarities between the two contexts.

### Translation challenges

In our attempts to translate Dutch data into English, we identified three dimensions of translation complexity that we will describe below: attending to grammar or syntax differences, grappling with metaphor, and capturing semantic or sociolinguistic nuances. We share examples from the study described above. When presenting quotes, it is common practice to identify which participants the quotes come from but, as the focus of this paper is on translation challenges instead of outcomes of the study, we do not refer to specific participants. Without access to the original interviews (raw data), it might be difficult for the reader to judge the quality of the translations. Again, following the purpose of this specific paper, we only use quotes to illustrate some of the challenges we encountered, not intending to provide a single or best final translation.

#### Attending to grammar or syntax differences

Although both Dutch and English are Germanic languages, we found differences in the use of grammatical elements such as tenses, articles, or nouns. When a student says in Dutch: ‘*Dus ehm, eh ja, zij is eigenlijk veel meer al aan het nadenken van nou, over* ***de dood***’ this would conceptually translate as ‘*So uhm, uhm yes, she is really even more, thinking of, well, about* ***death***.’ In Dutch, one uses an article (‘de dood’), but the literal translation of ‘the death’ would only been used in English to give specific emphasis, for instance ‘everyone hopes for the death of their choosing’.

Another example was the following phrase by one of the participants when talking about complex situations in medical education: ‘*dat je met meerdere eh* ***zorg*** *te maken hebt*’, which might translate as ‘*that you have to deal with multiple, uh,* ***nurses***.’ ‘Zorg’ is an abbreviation from ‘verzorging’, and is used here as a *pars pro toto, *indicating that something is named after a part of it. ‘Verzorging’ is a noun used quite often, which would literally be translated as ‘caregiving’ or ‘nursing’, but is commonly used, as in this quote, to refer to caregivers, i. e. the nurses or nursing staff. In a different context, the word ‘zorg’ could also be used to indicate the whole healthcare system.

#### Grappling with metaphor

When translating metaphors or idiomatic sayings, literal translations were unsatisfactory. Instead, we would endeavour to find a different, but conceptually equal metaphor. For instance, ‘*ze droomde eigenlijk al van een beetje* ***gaan hemelen***’, would literally be ‘*actually she already dreamed about* ***ascending into the heavens*** *a bit*’. However, taking into account the religious background of this patient and her wish to die, we decided that, as one of different possible translations, the nonliteral translation of ‘*she is ready to* ***meet her maker***’ better captured the intended meaning in English.

In other cases, we could not find an adequate metaphor in English, without losing the contextual meaning of the original phrase in Dutch. One of the students in the interviews talked about what is called ‘euthanasia’ in common Dutch, ‘termination of life on request and assisted suicide’ in legal Dutch and ‘physician-assisted death’, ‘physician-assisted suicide’ or ‘doctor-assisted dying’ in English. In the Netherlands, the *Termination of Life on Request and Assisted Suicide (Review Procedures) Act* took effect on 1 April 2002, legalizing euthanasia and physician-assisted suicide under very strict circumstances. After almost 15 years, however, the discussion about euthanasia in the Netherlands tends to be broadened to include patients who were previously excluded from euthanasia, for instance patients suffering from psychiatric diseases or dementia. Concerns are being expressed about what might become a ‘slippery slope’, and about doctors who may too easily grant euthanasia requests. This is the context the student refers to when saying: ‘*Dan ga ik ook nadenken van ja, euthanasie is ook maar iets alsof, alsof je, alsof het makkelijk* ***uit de muur te trekken*** *is*.’ A literal translation would be: ‘*Then I start thinking, yes, euthanasia is also just something, as if, if you could easily* ***pull it from the wall***’. A more conceptual translation might be to not use ‘pulling it from the wall’, but refer to euthanasia ‘*as if, if you could easily* ***grab it from a vending machine***.’ This translation tries to convey some of its contextual meaning, with its connotation with fast food, which in the Netherlands is often considered quick and dirty. Here, the professional backgrounds and cultural contexts of the researchers clearly influenced our interpretation of this particular phrase.

#### Capturing semantic or sociolinguistic nuances

Language is influenced by social and cultural factors, and translations should try to capture these semantic of sociolinguistic nuances. For example, we translated ‘*ondertussen hadden zij het gevoel dat wij hun moeder aan het* ***ombrengen*** *waren*’ into ‘*in the meantime, they felt we were* ***killing*** *their mother*.’ In Dutch, murder is ‘moord’, which is an intended crime and killing is ‘doodslag’, which does not need to be intentional. ‘Ombrengen’ is something else, closest to killing, but slightly softer, and in the context of healthcare, at least as expressed in the interview we took this quote from, it may better fit the notion of doing good to the patient, when the treatment may do more harm than good, but it still has the meaning of killing in it.

As the example of ‘ombrengen’ illustrates, context was a key dimension informing our translations. Attention to contextual nuance sometimes meant translating the same word differently in different situations. For instance, in the statement, ‘*pijnpatiënten zijn vaak patiënten waar je op een gegeven moment, eh, ja,* ***afkeer*** *is dan ook weer geen goed woord …*’, ‘afkeer’ was translated as ‘aversion’: ‘*pain patients often are patients, whom you, one time, uh, yes,* ***aversion*** *is not really a good word …*’. In a second quote, about a man who was suspected of having committed sexual offenses, we translated ‘afkeer’ into ‘disgust’. The Dutch quote was: ‘*Een* ***afkeer*** *denk ik. Ik heb het niet aan die man laten merken hoor maar dat is gewoon voor mezelf eh, voelde*.’ In English, this became: ‘***Disgust*** *, I guess. I didn’t show it to that patient, you know, but that is just what I, for myself, uh, what I felt*’. In these examples, we primarily built on the context described by the participant. Listening to the audio tapes, which EH did as well, can further inform the understanding of the intended meanings, but we think this is not unique to translations, but a more general analytic approach. At this point, we realized that a transcription is already a translation: from spoken language into written text.

In the last example given above, following the feeling of disgust, the participant used an understatement to express the opposite meaning: ‘*Dat ik af en toe dacht van, dat ik gewoon ja, een beetje een vies gevoel bij die man had, dat ik had* ***dat ik het niet erg vond als we weer weg gingen***.’ A literal translation would be: ‘*That is, sometimes, I thought, that I just, yes, had a bit of a dirty feeling with this man, that I had,* ***that I didn’t mind when we left again***.’ What the participant is really saying, however, is: ‘*That is, sometimes, I thought, that I just, yes, had a bit of a dirty feeling with this man, that I had,* ***I couldn’t get out of there fast enough.***’

These examples led us to question if and how the verbal syntax used by the participant, such as the ums and ahs, and the stops and starts, should be retained in the translation. Cleaning up such features in translation may improve readability, but when they create a tone of tentativeness that adds meaning in the original text, as in the examples above, it may be important to retain such features.

A final example shows that some words, notions or concepts cannot be translated literally in the target language, because they carry with them a host of contextual (cultural, legal, political) meanings. One of the students reflected: ‘*dat je vraagt of je goed hulp dat eh, dat je in strijd staat van belangen – is het* ***goed hulpverlenerschap?*** **’ **In the translation, we could capture the referential content as follows: ‘*That you are asking if you do right, helping, that uhm, that you are in a conflict of interests – is this* ***doing good as a professional*** *?*’ Although this may seem a rather adequate description of ‘goed hulpverlenerschap’, indicating what people are doing when they are doing good as a professional, it does not convey the specific (legal and ethical) aspects of the Dutch term. In the Netherlands, ‘goed hulpverlenerschap’ is a formal element of the ‘Act on Medical Treatment’, de ‘*Wet op de Geneeskundige Behandelovereenkomst*’, regulating the doctor-patient relationship.

### Relationship to the literature on translations

#### What is a valid translation?

Before discussing translations in specific stages of the research process, we want to address the question: ‘What entails a valid translation?’ With this question, we enter an epistemological debate [12]. Researchers within a positivist paradigm may strive for objectivity, trying to reach a ‘correct’ version of the text, for example by using professional translators and procedures such as forward-backward translation [8]. Scholars adopting a constructivist approach, however, will acknowledge that people who use different languages construct different ways of seeing social life, appreciating that there can be no single correct translation, and that both source and target language always mirror specific cultures and identities [13]. When engaging in translations, it is important to make sure that both semantic and conceptual equivalence are reached [14, 15]. Semantic equivalence is concerned with the transfer of meaning across languages, attending to whether words do really mean the same thing [16]. Linguists and translators distinguish between different types of meaning, such as referential meaning (the ideas or objects the word refers to), connotative meaning (the emotional response evoked by the word), social meaning (words that are used in a specific social context), or affective meaning (how words reflect the views and feelings of the speaker) [14]. An example of these nuances can be found in our struggles with translating the phrase about euthanasia, as we described above. Conceptual equivalence is reached when the words used have the same relationship to underlying concepts in both cultures [16]. One instance where we encountered difficulties in reaching conceptual equivalence was when we tried to translate *‘goed hulpverlenerschap’*, as we described above.

Constructivist scholars advocate to make translation visible as part of the research process, carried out in open dialogue by professional translators and/or researchers with a certain level of language competence, and to re-conceptualize translation as a practice rather than a product of language equivalence [17]. As a consequence, it is deemed critical to transparently report the roles and demographic characteristics of both researchers and translators, reflecting on how they may have influenced the construction of data [4, 10, 12].

There is consensus that a translator should have at least sociolinguistic competence [11]. In our own study, EH was the main translator and the only team member qualified according to these criteria.

#### Translating during analysis

When we started analysing our Dutch data, we considered whether the first stage of open coding should be in English or in Dutch. As the original text of the interviews and the first language of EH, Dutch as the language for coding seemed intuitively easier as a means of engaging with participants’ narrative reports in the interviews. However, since the vast majority of the scientific literature EH reads and writes is in English, and the rest of the research team could not engage with her open codes if they were written in Dutch, we decided that coding in all stages would be in English.

In particular when doing grounded theory research, but also within other methodologies, one should deliberately make choices about translations at different moments, going from initial coding, to intermediate coding and more advanced analysis [9]. Doing the initial coding in the original language may facilitate the response to what is going on in the text. This can be helpful for researchers who feel more comfortable in their own mother tongue, but may also be relevant as grammar and syntax vary across languages. However, there are also advantages to beginning translational efforts at the earliest stages of coding. Whereas initial coding in an inflectional language, such as Greek, Italian or Spanish, may be easier in the source language, the use of English, as a more analytic language, may facilitate abstraction [18].

Other structural differences between languages may also have implications for the choice of the coding language, such as the use of so-called gerunds (verbs becoming nouns, or ‘‑ing words’, e. g. dancing, doubting, dating). Grounded theory researchers may be familiar with the preferred use of those gerunds to indicate dynamic movement and emphasize social processes [19], but may not be aware of the fact that there is no equivalent of ‘gerund’ in several languages, for example in Indonesian [5] or Italian [18]. Our own study involved two different languages that both belong to the same (Indo-European) language family, and can both be considered rather analytic. However, gerunds are not as prevalent in Dutch as in English. Both the structural similarities between the two languages, and the possibility to use gerunds in English, supported our decision to do the coding in English.

Coding in another language can be considered a challenge, but also a resource, since it may enhance the interpretation, requiring continuous acts of translation, including decoding in the source language, and recoding in the target language. This continuing process of decoding and recoding in the two languages represents a powerful procedure that allows the researcher to make sense of the data [18].

#### Translating during writing up

When it comes to translating at writing up, the most important thing to realize may be that, generally, no reference is made to translation issues, as if it were unproblematic. Often data (quotes) are presented as if the participant was fluent in the target (English) language, which is not always (almost never!) the case [10]. Moving from one language into another and trying to have coherent texts in both, i. e. producing a normal-sounding or normal-reading text in the target language, implies making alterations of what was there in the original, requiring suppressions or additions [20]. Within anthropology, in respect for the otherness of the source text, some writers favour ‘ugly’ translations, that are not easy to read, being radically ‘sourcist’ and radically contextualizing but, as such, perfectly clear. Presenting both the original text and one or more possible translations, together with explanatory footnotes or comments, conveys a collaborative translation practice which seeks to really open up both a text and its world to the reader [20]. We have provided some examples of these ‘annotated’ translations above.

## Reflections

By sharing our experiences and providing some concrete examples, we hope to spark a critical discussion regarding the complexities of translation and help develop systematic strategies for qualitative scholars involved in multilingual medical education research projects.

When reflecting on our experiences with translations in an international qualitative research collaboration, the most important lessons we have learned are threefold. First, we realized that we, as a team and as individual researchers, had not been consciously aware of the ever-present need to translate in an international research community. This was clearly true for the two Canadian-born researchers. But even the other two members of the team, for whom English is not their first language, had never explicitly reflected on this issue before. Second, we came to appreciate that translation challenges arise not only from differences in language, but also pertain to cultural or societal differences regarding for example politics, economics, educational systems, and the organization and financing of healthcare. This relates to our third lesson, which is about exploring and celebrating cultural difference. We do acknowledge that the cultural differences between two Western, developed countries may seem relatively limited, in comparison to cross-cultural research including Latin-American, Arabian, or Asian cultures. However, by playing around with words and concepts, and by working (sometimes effortfully!) to convey to each other the nuanced, culturally flavoured meanings of notions, some differences turned out to be substantial and unexpected. Being reflective about translations thus proved enriching, rewarding, stimulating and inspiring, and from that angle, can be considered a promising avenue for further enhancing the field of medical education research.

Thus, as a conclusion, we would like to emphasize that researchers should be critical and conscious of the choices they make regarding translations in qualitative studies, throughout the different stages of the research process. This implies that researchers explicitly describe their translation strategies, most likely under the ‘methods’ heading of empirical research papers, and that they include some of their ‘ugly’ or ‘annotated’ translations in the manuscript. The choice for a specific translation strategy should mirror the epistemological position of the researchers, and as such, is likely to add to the trustworthiness and quality of the research, producing qualitative findings truly reflective of the participants.

## References

[1] Helmich E, Yeh H, Kalet A, Al-Eraky M (2016). Becoming a doctor in different cultures: towards a cross-cultural approach to supporting professional identity formation in medicine. Acad Med.

[2] Hodges B, Maniate J, Martimianakis M, Alsuwaidan M, Segouin C (2009). Cracks and crevices: globalization discourse and medical education. Med Teach.

[3] Bassnett S (2014). Translation studies.

[4] Squires A (2009). Methodological challenges in cross-language qualitative research: a research review. Int J Nurs Stud.

[5] Nurjannah I, Mills J, Park T, Usher K. Conducting a grounded theory study in a language other than English: procedures for ensuring the integrity of translation. Sage Open. 2014. doi:10.1177/2158244014528920.

[6] Epstein JS, Santo RM, Guillemin F (2015). A review of guidelines for cross-cultural adaptation of questionnaires could not bring out a consensus. J Clin Epidemiol.

[7] Kosny A, MacEachen E, Lifshen M, Smith P (2014). Another person in the room: using interpreters during interviews with immigrant workers. Qual Health Res.

[8] Chen H, Boore J (2009). Translation and back-translation in qualitative nursing research: methodological review. J Clin Nurs.

[9] Santos HJ, Black A, Sandelowski M (2015). Timing of translation in cross-language qualitative research. Qual Health Res.

[10] Al-Amer R, Ramjan L, Glew P, Darwish M, Salamonson Y (2014). Translation of interviews from a source language to a target language: examining issues in cross-cultural health care research. J Clin Nurs.

[11] Squires A (2008). Language barriers and qualitative nursing research: methodological considerations. Int Nurs Rev.

[12] Croot E, Lees J, Grant G (2011). Evaluating standards in cross-language research: a critique of Squires’ criteria. Int J Nurs Stud.

[13] Larkin P, Dierckx de Casterle B, Schotsmans P (2007). Multilingual translation issues in qualitative research: reflections on a metaphorical process. Qual Health Res.

[14] Herdman M, Fox-Rushby J, Badia X (1998). A model of equivalence in the cultural adaptation of HRQoL instruments: the universalist approach. Qual Life Res.

[15] Bowden A, Fox-Rushby JA (2003). A systematic and critical review of the process of translation and adaptation of generic health-related quality of life measures in Africa, Asia, Eastern Europe, the Middle East, South America. Soc Sci Med.

[16] Beaton DE, Bombardier C, Guillemin F, Bosi Ferraz M (2000). Guidelines for the process of cross-cultural adaptation of self-report measures. Spine.

[17] Wong J-H, Poon M-L (2010). Bringing translation out of the shadows: translation as an issue of methodological significance in cross-cultural qualitative research. J Transcult Nurs.

[18] Tarozzi M (2013). Translating and doing grounded theory methodology. Intercultural mediation as an analytic resource. Forum Qual Soc Res.

[19] Charmaz K (2006). Constructing grounded theory: a practical guide through qualitative analysis.

[20] Leavitt J (2014). Words and worlds ethnography and theories of translation. Hau J. Ethnogr. Theory.

